# Sunflowers as an Antiperiodic

**Published:** 1890-09-13

**Authors:** 


### SUNFLOWERS AS AN ANTIPERIODIC.

This plant has from time immemorial been a popular remedy in malarial fevers throughout Russia, Turkey, and Persia, in the former country being employed in the form of a tincture made by steeping the stems and leaves in brandy. But the quantity of alcohol in the popular preparation being excessive, Dr. Filatoff, of Moscow, recommends one made with sp. vin. rect., and one fifth by weight of the fresh plant, the dose for an adult being then about half an ounce, still too much we should think to be taken three times a-day. From a wide experience of its use he considers it even preferable to quinine, being at least as efficacious in all cases, succeeding when quinine had failed, and being free from any of the inconveniencies incident to large doses of that drug. Not only do the most inveterate cases of intermittent fever yield to it, but the appetite and general health improve, and the patients rapidly gain in strength and weight. It was first introduced into scientific medicine by Dr. P. Filatoff, of Sarausk, in 1879, and his favourable reports were later confirmed by Drs. Kazatchek and Maminoff, of Moscow. Dr. N. Filatoff now recommends it strongly for all types of malaria in persons of all ages, its pleasant taste being no small advantage in the case of children. Cases in which both quinine and arsenic had proved useless have in his practice been completely cured in a week or ten days.

